# Individual differences in COVID-19 mitigation behaviors: The roles of age, gender, psychological state, and financial status

**DOI:** 10.1371/journal.pone.0257658

**Published:** 2021-09-21

**Authors:** Joel Myerson, Michael J. Strube, Leonard Green, Sandra Hale

**Affiliations:** Department of Psychological & Brain Sciences, Washington University in St. Louis, St. Louis, MO, United States of America; La Sapienza University of Rome, ITALY

## Abstract

The present study examined individual characteristics potentially associated with changes in mitigation behaviors (social distancing and hygiene) recommended by the Centers for Disease Control and Prevention. Analysis of online survey responses from 361 adults, ages 20–78, with US IP addresses, identified significant correlates of adaptive behavioral changes, with implications for preventive strategies and mental health needs. The extent to which individuals changed their mitigation behaviors was unrelated to self-rated health or concern regarding the personal effects of COVID-19 but was related to concern regarding the effects of the pandemic on others. Thus, mitigation behaviors do not appear to be primarily motivated by self-protection. Importantly, adaptive changes in mitigation behaviors increased with age. However, these changes, particularly those related to the frequency of close proximity encounters, appear to be due to age-related decreases in anxiety and depression. Taken together, the present results argue against over-reliance on ‘fear appeals’ in public health messages as they may increase anxiety and depression. Instead, the present findings argue for more appeals to people’s concern for others to motivate mitigation as well as indicating an immediate need to address individual mental health concerns for the sake of society as a whole.

## Introduction

Older adults infected with the COVID-19 virus are at greater risk of severe complications, as reflected in the higher rate at which they require hospitalization [1] and the greater likelihood that they will die as a result of their infection [2], a finding that has been replicated in a sample of over 17 million patients [3]. Age is not the only factor associated with greater-than-average risks from COVID-19. Being male, for example, also appears to be associated with the risk of serious complications and even mortality, with poverty being another notable risk factor [3]. Of special interest from a public health perspective are behaviors that either increase the risk (close proximity interactions or actual physical contact with non-household members) or decrease the risk (disinfecting one’s hands and commonly used surfaces) of contracting COVID-19 and that could be appropriately modified.

With respect to age, previous studies suggest that its relation to perceived risks associated with COVID-19 are complicated. For example, the perceived risk of death from COVID-19 increases with age (e.g., mortality risk), but the perceived risk of being infected decreases with age as does the perceived risk of running out of money [4, 5]. It is unclear how these risk perceptions combine to affect mitigation behaviors, particularly in older adults, although structural equation modeling suggests that the perceived effectiveness of mitigation behaviors and not perceived COVID-19 risks predicts mitigation behaviors [6].

With respect to gender, there is as yet no consensus on mitigation behaviors. For example, men have been reported to be less likely to wear a face mask [7], but a failure to find gender differences in mask wearing also has been reported [8]. Women are more likely to get annual vaccinations for influenza [9], a mitigation behavior, but whether this difference holds for COVID-19 mitigation behaviors is unclear. To date, published studies have examined vaccination intentions and, perhaps surprisingly, men are more likely than women to intend to get vaccinated [10, 11]. Whether more men than women actually get vaccinated is yet to be determined, and in any case, the implications for gender differences in other mitigation behaviors are not yet established.

Concerns about the financial impact of the pandemic tend to decrease with age, perhaps because older adults are more likely to be retired and their income would be less affected [4]. Financial status appears to play a role in mitigation behaviors. For example, those with higher household incomes report being more likely to wear a face mask [7] and are less likely to show increased levels of psychological distress [12], raising the possibility that distress may be associated with mitigation.

The current study focuses on those mitigation (risk-reducing) behaviors recommended by the Centers for Disease Control and Prevention (CDC). We examined state characteristics of individuals because by definition, state variables are more likely to be modifiable than traits [13], and thus might provide targets for interventions. If people high in trait anxiety were more likely to engage in social distancing, for example, one would not be able to increase the trait anxiety of others low on this trait in order to promote such behavior, whereas if people high in state anxiety were the ones more likely to engage in social distancing, then one might try to raise everyone’s level of state anxiety. Importantly, the state/trait distinction has implications for public health messaging, because individual differences in trait anxiety are not predictive of the efficacy of fear messages (e.g., messages emphasizing risk), whereas such messages may well increase state anxiety, and indeed, may be intended to do so [14].

Accordingly, the present study analyzed the data from an online sample of 361 adults ranging in age from 20 to 78 years with US IP addresses and examined relations among self-reports of the frequency of four CDC-recommended mitigation behaviors and demographic variables and several measures of participants’ psychological state. These included the Hospital Anxiety and Depression Scale (HADS), which measures state anxiety and depression and has shown the ability to track changes in these states [15], as well as a new pandemic questionnaire we developed that includes items assessing concerns about potential effects of the COVID-19 pandemic on oneself and others.

Our pandemic questionnaire also measured self-reported frequencies of four CDC-recommended mitigation behaviors that concerned both social distancing (i.e., not being within six feet of someone outside one’s household and avoiding physical contact with such a person) and hygiene (i.e., washing or disinfecting one’s hands and disinfecting frequently touched objects and surfaces). The frequencies of mitigation behaviors are especially important because they represent potentially modifiable risk factors. In addition, the questionnaire included items concerning self-rated health and subjective wellbeing. Notably, the present study also examined the roles of gender and financial status in mitigation.

The present findings regarding correlates of mitigation behaviors have implications for our understanding of the psychological effects of the pandemic as well as our understanding of those mitigation behaviors believed to be most likely to affect the course of the pandemic itself. These findings also may have important implications for the formulation of effective public health messaging during the COVID-19 pandemic. For example, fear messages appear to work better when what is recommended is a one-time response, rather than a recurring activity as is the case with the mitigation behaviors recommended by the CDC [16], whereas messages emphasizing benefits to the community may work better for behaviors that need to be maintained. Examination of the relations between age and mitigation behaviors, on the one hand, and anxiety and depression, on the other hand, should shed light on the potential effects of increasing people’s state anxiety with fear messages and on the utility of this approach with respect to the current public health situation.

## Materials and methods

### Participants

This research was approved by the Institutional Review Board of Washington University in St. Louis, #201806131. The data were reported anonymously. MTurk workers were recruited online in two waves [17, 18]. The first wave, which was open to participants 18 and older, was on April 13, 2020 (N = 299, M_age_ = 36.7, SD = 11.1, Range = 20–67, 44.1% Female) and the second, which was intended to provide better representation of older adults in our sample (N = 71, M_age_ = 62.9, SD = 8.1, Range = 26–78, 71.8% Female), was recruited one week later. Although our goal was to recruit only those 55 and older, two younger women in their 20s also participated in the second wave. At the time the survey was administered, almost all 50 states had stay-at-home orders in place. Although the specifics of those orders varied, the timing of the study minimized effects of geographical and political variations in the constraints on people’s socializing, and the data reflect individual differences in participants’ compliance with their state’s orders and with CDC recommendations.

After indicating their informed consent online, which initiated the survey, a total of 370 MTurk workers provided their responses and received $1.00 for their participation, which took 11.6 min on average. The submitted surveys were then screened for age, valid IP addresses associated with internet providers in the United States of America, and survey completion time so as to exclude those whose times were less than the time a fast, expert reader would require to read the survey questions [19]. Based on these criteria, data from 9 individuals were excluded from our analyses, 2 based on invalid US IP addresses and 7 based on their completion times, leaving 361 participants, 181 female and 180 male, ranging in age from 20 to 78 years. Perhaps not surprisingly, given the greater longevity of women than men in the US, the proportion of female participants increased with age, but the age ranges of the female and male participants were nearly equivalent (20–73 and 21–78 years, respectively).

The racial/ethnicity breakdown of the participants was 81.4% White, 7.2% Asian, 6.9% Black, and 4.4% other races, and 20.2% identified as Hispanic/Latinx. Participants reported individual incomes ranging from 0 to 500,000 dollars per year and household incomes ranging from 0 to 700,000 dollars per year, with median incomes of $37,500 and $56,500, respectively. Their median years of schooling was 16; notably, 87.7% of participants had more than 12 years and 65.9% had completed 16 or more years. With a sample size of 361, we can detect a Spearman correlation as low as .15 with power of .80 and *p* = .05, two-tailed.

### Procedure

The online survey began with items from the IPIP-NEO (International Personality Item Pool-Neuroticism, Extraversion, Openness) personality test, the Hospital Anxiety and Depression Scale (HADS), and the Personal Need for Structure (PNS) scale. The order in which the three were presented was randomly determined. These were followed by a question concerning overall health (“At the present time, my overall health is: Excellent, very good, good, fair, poor”) and our brief, three-part Pandemic Questionnaire (see S1 Questionnaire).

The first part presented questions about the frequency of four CDC-recommended mitigation behaviors. Specifically, the four behaviors referred to two Social Distancing behaviors and two Hygiene behaviors: (1) being less than six feet from a person who was not a member of one’s household, (2) making physical contact with such a person, (3) cleaning one’s hands with either sanitizer or soap and water, and (4) disinfecting frequently touched objects and surfaces. These measures are labelled Proximity, Contact, Hand hygiene, and Home hygiene, respectively. The frequency of each behavior was investigated in two separate time frames, namely “on average this week” and “several months ago,” which, it should be noted, was mid-February, 2020, at the latest). Scores could range from 1 (never) to 5 (highest frequency). Also, measures of change in frequency (Now minus Before) were calculated for each of the four mitigation behaviors assessed. The symbol Δ indicates that a measure represents change in the frequency of a mitigation behavior. The second part of the Pandemic Questionnaire consisted of questions as to participants’ degree of concern about the effects of the pandemic on themselves and on others, and the third part asked questions regarding participants’ subjective wellbeing at the present time, two months previously, two months from the current time, and two years from the current time. Finally, participants were asked their gender, their age and date of birth (for verification), how many years of education they had completed, their ethnicity and race (as requested by the National Institutes of Health), and their annual individual and household income and zip code.

Data from the two scales that assessed personality traits (i.e., the IPIP-NEO and PNS) were not analyzed for the current effort, as they are not relevant to the scope of this study, which focused on measures of behavior and of psychological state. The HADS was originally developed to identify anxiety and depression among patients in non-psychiatric hospital clinics [20] and has since been shown to be suitable for online administration [21]. It is divided into Anxiety and Depression subscales, with the seven items of each subscale intermingled. The HADS has been found to perform well in assessing symptom severity in the general population as well as in medical patients [22], and it also has the ability to track changes in symptoms [15, 23], indicating that it measures state as well as possibly trait aspects of anxiety and depression.

### Data analysis

All analyses were based on data from 361 participants except for those involving Household Income, which was not reported by 22 participants. Follow-up analyses concerning anxiety and depression focused on the 211 participants whose scores on both the anxiety and depression scales of the HADS were below cutoffs for anxiety and depressive disorders based on frequencies of these disorders reported in large epidemiological studies [24].

Nonparametric statistical analyses were used because the mitigation raw scores and the change measures were categorical (albeit ordinal) in nature. Differences were assessed using Signed-Rank (Now vs. Before mitigation frequencies) and Rank-Sum tests (gender differences in mitigation). Observed differences between female and male participants in change in the frequency of close proximity encounters led to follow-up analyses to explicate these differences, including correlational analyses assessing associations between Age and change in the proximity measure in females and males separately using Spearman’s rho. We assessed associations between the 4 mitigation change measures and variables hypothesized to be associated with mitigation change using Spearman’s rho because of the categorical nature of our mitigation measures. In contrast to the preceding analyses, which examined the absolute size of changes in the frequency of mitigation behaviors, a final set of analyses examined the frequency with which changes were in the direction recommended by the CDC.

For the whole sample, applying a Bonferroni correction for multiple comparisons in the case of each mitigation behavior reduced the significance level to *p* < .004. Similarly, applying a Bonferroni correction to analyses of the associations of (binary) measures of whether or not CDC-recommended adaptive changes had occurred in mitigation behaviors with five non-mitigation variables lowered the significance level for these analyses to *p* < .010. Both the uncorrected probabilities and whether the observed probabilities were less than the corrected significance level are reported.

## Results

**Fig 1:**
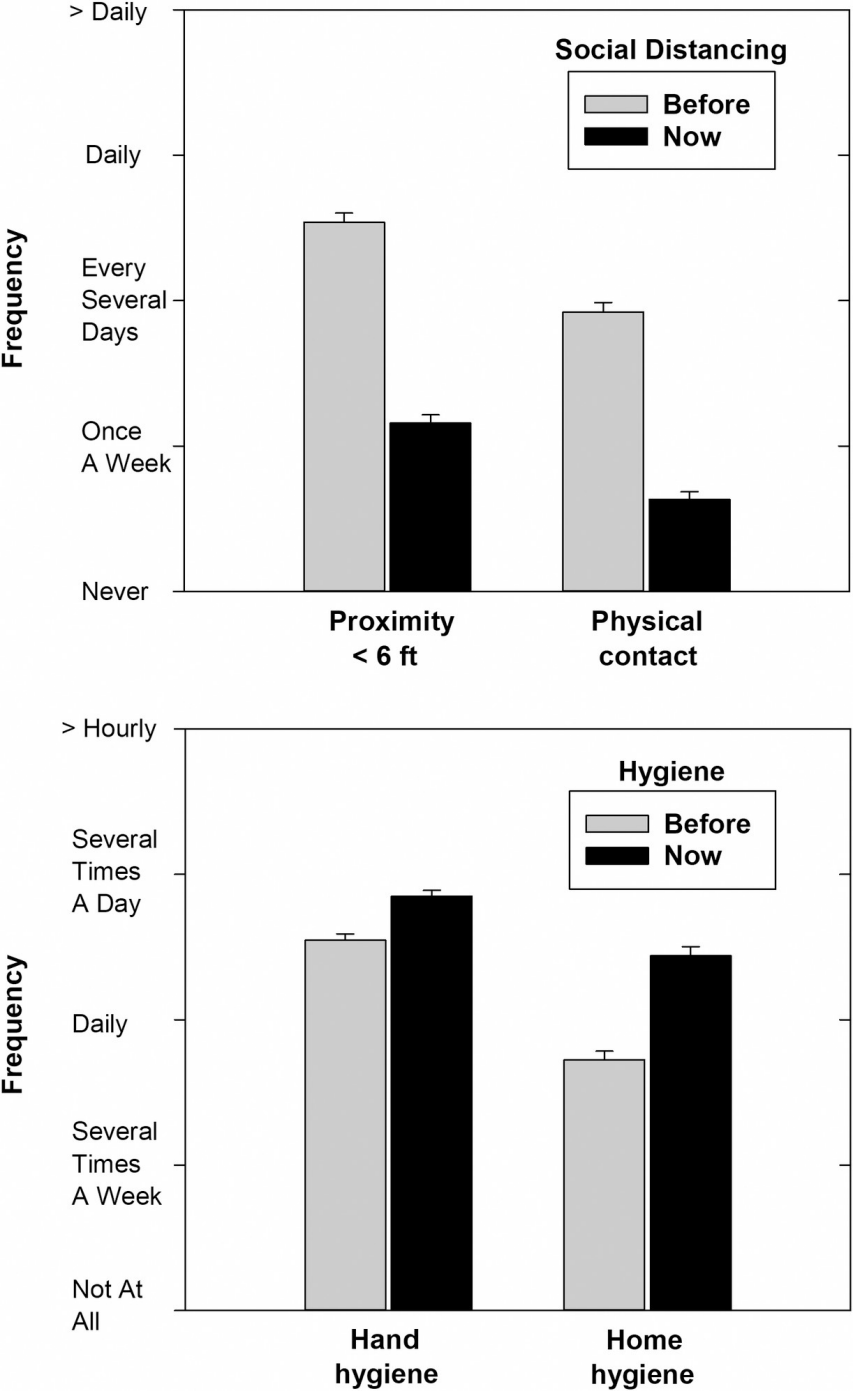
**Mean frequencies of two social distancing mitigation behaviors (upper panel) and two hygiene mitigation behaviors (lower panel) before and during the COVID-19 pandemic.** Error bars represent standard errors of the mean.

Compared with their behavior around the beginning of 2020, by mid-April participants had decreased the frequency with which they were less than six feet from a person outside their household as well as the frequency of physical contact with such person (Fig 1, upper panel). For example, participants reported that, on average, they had reduced the frequency of close proximity encounters from about once every couple of days before the pandemic to once a week. In addition to decreasing the frequency of physical contact, participants also reported that they had increased the frequency with which they cleaned their hands with sanitizer or soap and water and disinfected frequently touched objects and surfaces (Fig 1, lower panel). Signed-rank tests on the difference between the frequency of the four mitigation behaviors in mid-April during the pandemic and several months earlier before the pandemic was widely recognized in the U.S. were all significant (Table 1).

**Table 1:** Results of signed-rank tests comparing behavior before and during the pandemic.

Now—Before	W	*p*	Effect Size
**Proximity**	36,005.5	< .001	0.857
**Contact**	33,841.5	< .001	0.850
**Hand hygiene**	3,434.0	< .001	0.516
**Home hygiene**	3,108.0	< .001	0.732

Note. Effect size = Rank-Biserial correlation

To measure the amount of change in each mitigation behavior at the individual level, the score before the pandemic was subtracted from the score during the pandemic (Table 2). These change scores could range from -4 (maximum decrease in frequency) to +4 (maximum increase). The resulting four change measures were all significantly correlated (all *p*s < .001), the two social distancing measures strongly so (rho = .616). The two hygiene change measures, however, were not more strongly related to each other than they were to the distancing measures. Notably, the correlations between distancing and hygiene measures were all negative, indicating that participants who had decreased the frequency of close proximity encounters and physical contact with those outside their household also tended to have increased the frequency of hand and home hygiene behaviors (Table 3).

**Table 2:** Descriptive statistics for measures of change in mitigation behaviors.

	Distancing		Hygiene	
	Δ Proximity	Δ Contact	Δ Hand	Δ Home
Mean	-1.38	-1.29	0.30	0.72
Stand. Dev.	1.56	1.52	0.89	1.23
Median	-1.0	-1.0	0.0	0.0
Q1	-3.0	-2.0	0.0	0.0
Q3	0.0	0.0	1.0	2.0
Min	-4.0	-4.0	-3.0	-4.0
Max	3.0	4.0	3.0	4.0

**Table 3:** Intercorrelations among mitigation change measures.

		Δ Proximity	Δ Contact	Δ Hand hygiene
**Δ Proximity**	rho	--		
	*p*			
**Δ Contact**	rho	.616	--	
	*p*	< .001		
**Δ Hand hygiene**	rho	-.196	-.213	--
	*p*	< .001	< .001	
**Δ Home hygiene**	rho	-.289	-.341	.200
	*p*	< .001	< .001	< .001

**Fig 2:**
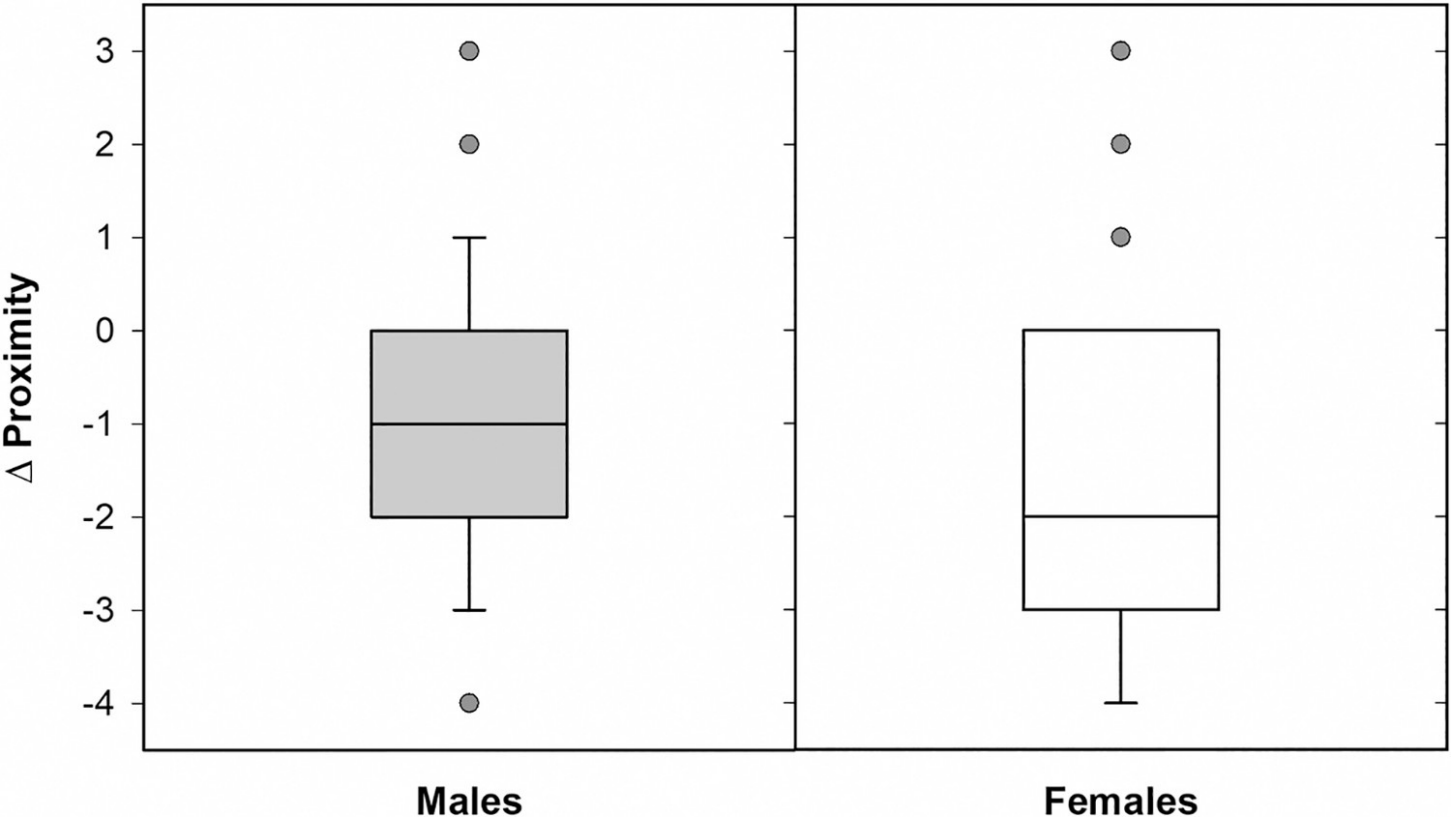
Box plots of the degree of change in female and male participants’ close proximity encounters since the beginning of the COVID-19 pandemic.

**Fig 3:**
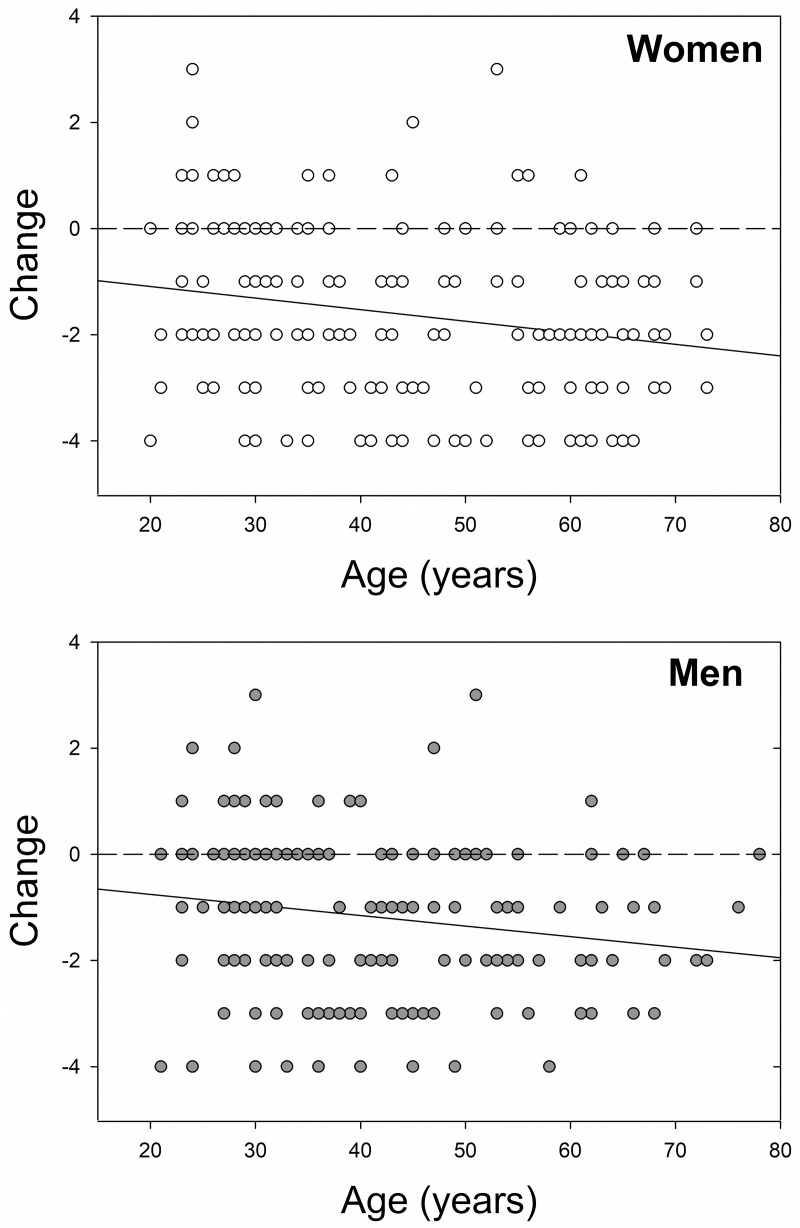
**Change in frequency of close proximity encounters (ΔProximity) as functions of age for female (top panel) and male (bottom panel) participants.** Note that because of the categorical nature of our frequency measure, some symbols represent data from more than one participant.

Women and men reported equivalent changes in Contact, Hand hygiene, and Home hygiene (Table 4). In contrast, women showed larger differences than men between the frequency with which they were less than six feet from another person outside their household before versus during the pandemic (Fig 2). Follow-up analyses revealed that this was entirely due to a gender difference in the frequency of such encounters during the pandemic (Mann Whitney U = 13,367.5, *p* = .002), as women reported having slightly (but not significantly) more close proximity encounters than men before the pandemic (U = 15,412.0, *p* = .360). Further analyses conducted to explicate the gender difference in Proximity revealed that although women overall reported larger changes in Proximity, both women’s and men’s change scores became significantly more negative with Age (rho = -.227, *p* = .002, and rho = -.253, *p* < .001, respectively; Fig 3).

**Table 4:** Results of rank-sum tests of gender differences in changes in mitigation behaviors.

	U	df	*p*	Effect Size
**Δ Proximity**	13,399.0	359	.003	0.177
**Δ Contact**	15,182.0	359	.253	-
**Δ Hand hygiene**	16,234.0	359	.951	-
**Δ Home hygiene**	17,379.5	359	.251	-

Note. Shapiro-Wilk tests revealed significant departures from normality; all Ws > .80, ps < .001. Effect size is given by the rank-biserial correlation.

Age was also significantly associated with change in the other social distancing measure (i.e., the frequency of physical contact) which also decreased with age, but not with changes in either hand or home hygiene (see *Risk factors* in Table 5). None of the correlations of self-rated health with the four mitigation behaviors was significant following Bonferroni correction.

**Table 5:** Correlations of changes in mitigation behaviors with potential associates.

		Δ Distancing		Δ Hygiene	
		Proximity	Contact	Hand	Home
***Risk factors***					
**Age**	rho	-.249	-.222	.091	.127
	*p*	< .001*	< .001*	.086	.016
**Health**	rho	.092	.029	-.034	-.116
	*p*	.081	.579	.515	.027
***Income and education***					
**Individual**	rho	-.038	-.050	.008	-.033
	*p*	.470	.343	.875	.531
**Household**	rho	-.155	-.138	.045	.017
	*p*	.004*	.011	.405	.749
**Education**	rho	-.010	.034	-.069	-.023
	*p*	.847	.526	.194	.665
***Psychological state***					
**Concern: Self**	rho	-.044	.020	.039	.149
	*p*	.402	.709	.465	.005
**Concern: Others**	rho	-.170	-.126	.079	.165
	*p*	.001*	.017	.132	.002*
**Depression**	rho	.305	.252	-.071	-.101
	*p*	< .001*	< .001*	.177	.056
**Anxiety**	rho	.373	.296	-.056	*-*.171
	*p*	< .001*	< .001*	.285	.001*
**Well-being now**	rho	.052	-.070	-.026	-.040
	*p*	.321	.183	.619	.447
**Well-being in 2 yrs**	rho	.084	.059	.048	.027
	*p*	.114	.267	.366	.612

Note. * Indicates p < the Bonferroni-corrected significance level.

There was a significant negative correlation between household income and **Δ**Proximity (see *Income and Education* in Table 5) indicating that the greater the household income, the more negative the difference between Proximity “now” and “before.” However, neither individual income nor education was associated with the degree of change in any of the mitigation behaviors (all *p*s > .193).

Notably, participants’ level of concern regarding the effects of the pandemic on others was positively associated with CDC-recommended changes in both close proximity encounters and home hygiene, whereas concern for the personal effects of the pandemic was not (see *Psychological state* in Table 5). Both depression and anxiety, which were positively correlated in the current sample (*r* = .670), were also positively correlated with social distancing measures. This reflects the fact that higher scores on depression and anxiety were associated with smaller decreases in the frequency of distancing behaviors, resulting in change scores that tended to be less negative. Moreover, the greater the level of anxiety, the smaller the increase in home hygiene. Neither participants’ satisfaction with their current life nor their expected future life satisfaction was associated with changes in their mitigation behaviors.

### Age, psychological distress, and social distancing

Both anxiety and depression decreased with age (both rhos < -.21, both *p*s < .001), which could have contributed to the association of age with appropriate changes in mitigation behaviors, particularly social distancing. To explore this possibility, we focused on the 211 participants whose anxiety and depression scores were below empirically based cutoffs for anxiety and depressive disorders [24]. Among those for whom neither score was above the caseness cutoff, **Δ**Proximity was not significantly related to age (rho = -.108, *p* = .117), although **Δ**Contact was (rho = -.147, *p* = .033), albeit at a lower level than in the sample as a whole.

### Adaptive change

Whereas the preceding analyses focused on what variables are associated with the amount of change in the four CDC-recommended mitigation behaviors, the present analyses focused on whether or not adaptive changes (i.e., decreases in the social distancing measures and increases in the hygiene measures) had occurred, regardless of the size of those changes, as well as on how many of a participant’s mitigation behaviors showed adaptive changes. The reason for examining whether or not adaptive changes had occurred was to determine whether there was converging evidence for those variables identified in the preceding analyses as associates of change in mitigation behaviors and to estimate which of those variables might provide the biggest return from a public health perspective because they were associated with the most changed behaviors. Only about one-third (35.5%) of the participants reported an adaptive change in hand hygiene, and none of the 13 variables studied was significantly associated with changes in hand hygiene in previous analyses, perhaps because such changes were obscured by the frequency of handwashing before eating and after using the toilet. Therefore, hand hygiene was not included in any further analyses.

In contrast to the results for hand hygiene, approximately half of the participants reported adaptive changes in home hygiene (49.0%), and approximately two-thirds reported adaptive changes in the frequency of close proximity encounters and physical contact (67.3% and 65.1%, respectively). Taken together, approximately four-fifths (82.0%) of the participants reported an adaptive change in at least one of these mitigation behaviors. Accordingly, the present analyses focused on the five variables that, after correcting for multiple comparisons, had been identified as significant correlates of the amount of change in social distancing and home hygiene: Age, Concern for Others, Depression, Anxiety, and Household Income. All of the variables except Concern for others were related to whether or not the two social distancing measures changed, but only Concern for Others was associated with the occurrence of adaptive change in Home Hygiene. Importantly, all five of these previously identified variables were correlated with the total number of mitigation behaviors by a participant that showed adaptive changes (Table 6). Analogous to the findings with respect to amount of change, the fact that the correlations with Anxiety and Depression were again negative meant that the higher the participants’ scores on these scales, the smaller the number of adaptive changes in mitigation behaviors they reported.

**Table 6:** Correlations of five independent variables with adaptive changes in mitigation behaviors.

		Others^1^	Depression	Anxiety	Age	Income^2^
**Δ Proximity**	rho	.134	-.316	-.333	.251	.145
	*p*	.011	< .001*	< .001*	< .001*	.008*
**Δ Contact**	rho	.057	-.185	-.239	.151	.154
	*p*	.277	< .001*	< .001*	.004*	.005*
**Δ Home** ^3^	rho	.137	-.075	-.113	.041	.041
	*p*	.009*	.154	.032	.435	.455
**Δ** Total^4^	rho	.151	-.240	-.290	.190	.146
	*p*	.004*	< .001*	< .001*	< .001*	.007*_

^1^ Concern for Others

^2^ Annual Household Income

^3^ Home hygiene

^4^ The total number of behaviors that showed adaptive changes.

* Indicates *p* < the Bonferroni-corrected significance level.

## Discussion

The majority, but far from all, of the participants in the present study followed CDC recommendations for mitigating the effects of the COVID-19 pandemic. Most notably, participants significantly increased their social distancing, which is to say that they decreased the frequency of close proximity encounters with people other than those they lived with and also decreased the frequency of physical contact with anyone outside their household. Importantly, participants’ age, which is known to be a major risk factor for hospitalization, serious complications and even death from a COVID-19 infection [1, 3, 25], was significantly associated with the degree of adaptive change in both social distancing behaviors.

Changes in social distancing behaviors were positively correlated with anxiety and depression, with participants who had higher levels of anxiety and/or depression being both less likely to have decreased the frequency of close proximity encounters and physical contact with people outside their household members and to have made smaller changes if they did increase their social distancing. Consistent with previous reports [26], however, anxiety and depression in the current sample were generally rather high and decreased with age, raising the possibility that the reason older adults were more likely to make larger adaptive changes in their mitigation behaviors was because they were less anxious and depressed. Further analysis yielded results consistent with this interpretation. When the sample was divided into those whose anxiety and depression scores were below cutoffs for anxiety and depressive disorders and those with at least one score above the cutoffs, adaptive change in the frequency of close proximity encounters was not significantly related to age in the former group (i.e., those with scores below the cutoffs).

Is it possible that the reported changes in mitigation behaviors primarily reflect the social desirability of these changes, all of which were frequently advocated in public health messaging at the time of the survey? Although social desirability might play some role in both self-reports and actual behavior change, the fact that self-reported changes were much larger for distancing than for hygiene, combined with the low correlations between all but the two distancing measures (see Table 3), argue against general social desirability as an explanation for the reported changes in mitigation behaviors. We believe that distancing changed much more than hygiene because social deprivation not only leads to loneliness but also causes increases in anxiety and depression, which were significantly correlated in the present study, and social interaction relieves the stress caused by social deprivation [27, 28].

The present results suggest that more anxious and depressed people are choosing to alleviate or at least not aggravate their anxiety and/or depression by distancing. Further evidence for the idea that high anxiety and depression scores are the result of social deprivation comes from a study that examined anxiety levels as measured by the Generalized Anxiety Disorder-2 screening tool [29] used in the National Health Information Survey and the Understanding America Study. The proportion of participants with high levels of anxiety symptoms had more than doubled from their 2019 levels by the beginning of April, 2020, when stay-at-home orders were in effect in most states. When many states ended such restrictions in May, anxiety levels immediately dropped to near half their April levels, still higher than 2019 but much lower than during the stay-at-home period. Notably, although both case and death rates also declined [26], they did so to a much smaller extent than anxiety. Thus, the decrease in anxiety suggests that it was social isolation that had been responsible for the previous high levels. It may be noted, however, that the percentage of participants in the Household Pulse Survey who reported anxiety symptoms showed a much smaller, but still significant decrease from late April to mid-May of 2020 [26].

Higher household income was associated with increased social distancing, but neither education nor individual income, both of which tend to be higher for higher-status occupations, were significant associates, although the absence of significant correlations with education may reflect restriction of range (approximately 85% had some college and almost 2/3 had at least 4 years). Those with lower household incomes were less likely to decrease the frequency of close proximity encounters, but the fact that individual income was not correlated with distancing, whereas household income was, suggests that an individual’s financial resources are more important than their occupation in providing the opportunity to practice social distancing. In addition, it has been argued that lack of resources of any kind, not just financial resources, induces a ‘scarcity mindset’ that causes people to focus more on decisions relevant to the scarce commodity and to neglect other problems [30]. Scarce financial resources, for example, may lead to the neglect of health-related issues such as those raised by the pandemic, but the same may apply to scarcity of social interactions, with the same result.

It has been reported that those with lower household incomes are less likely to wear face masks [7], a mitigation behavior that had not yet been recommended by the CDC at the time of this study, and their jobs are likely to be less conducive to social distancing. The present results suggest that the relation of household income to mitigation extends beyond its association with mask wearing, and as already noted, the relation of income to psychological distress may contribute to its role in mitigation.

The lower frequency of social distancing behaviors among those with lower household incomes is especially unfortunate because poverty has been shown to predict the risk of dying if one is infected with the COVID-19 virus, independent of one’s pre-existing disease and other clinical risk factors [3]. Moreover, the observed association of household income and social distancing suggests that providing financial assistance during the pandemic constitutes a public health intervention because it could benefit not just those receiving the assistance but also may benefit society as a whole by facilitating mitigation behaviors that help prevent spread of the COVID-19 virus.

In the present study, gender differences were observed with respect to only one mitigation behavior, close proximity encounters. To date, the evidence regarding gender differences in mitigation has been mixed. For example, women have been reported to be more likely to wear a mask but no difference between men and women in this regard has also been reported [8, 7]. Women tend to be more likely to get vaccinations not only for influenza, but other diseases as well [9, 31]. Nevertheless, they have been reported to be less likely to plan to be vaccinated for COVID-19 [10, 11]. It will be interesting to see who actually gets vaccinated, but the relation of gender to mitigation, like that of age to pandemic risk perception, appears to be complicated.

Overall, the strongest associations observed between possible risk factors and mitigation behaviors were the relations of social distancing to anxiety and depression: The more anxious or depressed participants were, the less likely they were to have changed their behavior as recommended by the CDC, and if they did change their behavior, the resultant change tended to be smaller than the changes reported by those who were less anxious or depressed. This finding is important because of its implications for public health messaging. The use of ‘fear appeal’ messaging in public health communications (e.g., health warnings on cigarette packages) has a long history of mixed results [32], and the mix of successes and failures with such messages is frequently attributed to people having both adaptive and defensive responses to such messages [14]. Denial, for example, is a prominent defensive response that competes with more adaptive responses and could have interfered with people adjusting their behavior to reduce the risks from the COVID-19 pandemic.

One potential problem with fear messages, even accurate ones, is that they may make people anxious or depressed, and the present findings suggest that anxiety and/or depression can interfere with adaptive responses such as those recommended by the CDC. Many participants in the present study presumably were exposed to frequent fear messages, often well-intentioned, from various sources including the news media, as well as to conflicting messages. This combination may have created uncertainty and confusion in addition to anxiety and/or depression, ultimately leading to the observed deficiency in adaptive responses (e.g., social distancing) of those who were most sensitive.

Indeed, the high levels of anxiety and depression reported by participants suggest that most people are now aware of the risks associated with the pandemic. Thus, further ‘fear’ messages may have little informational value except, of course, when new information does come to light, a case in point being the emerging consensus that face masks may protect the wearers. Given the updated guidelines from the CDC, further research on the determinants of mask-wearing would be highly desirable, as would larger samples that include even older adults. Nevertheless, the present findings concerning anxiety and depression levels echo those from very large, nationally representative samples, suggesting that the present results may be representative as well. For example, anxiety and depression scores on the HADS decreased with age, in keeping with the results of the national Household Pulse Survey [26].

An important question is whether participants’ state or trait anxiety affected their tendency to mitigate pandemic risks. Indeed, participants’ scores on the HADS, which indicated their level of anxiety and depression, may reflect traits as well as states induced by the pandemic. However, a meta-analysis of the literature on fear appeals indicated that trait anxiety does not affect responses to fear appeals [14]. Thus, it is likely that it was their state, not trait, anxiety (and depression) that underlay the observed decrease in the likelihood and magnitude of adaptive mitigation behaviors in those with higher HADS scores.

Emphasizing the efficacy of adaptive responses can counteract defensive responses to fear messages [14]. However, the results of another meta-analysis [33] suggest that people’s belief that recommended behaviors are highly effective is critical, and currently there are obstacles to achieving such belief on the scale required by the COVID-19 pandemic. Moreover, fear messages appear to work better when what is recommended is a one-time response, rather than a recurring activity as is the case with the mitigation behaviors recommended by the CDC [16].

Fortunately, the present findings suggest another, complementary approach. Notably, although concern about the personal effects of COVID-19 was not associated with changes in any mitigation behaviors, concern for others was. A pre-pandemic study in a hospital setting provides an example of application of this finding to public health messages on a small scale: Health-care professionals increased their hand hygiene in response to a message highlighting the benefits to patients but not in response to one highlighting the benefits to themselves [34]. Evidence that altruistic appeals might motivate mitigation behavior in nonmedical personnel on a large scale comes from survey responses during the Severe Acute Respiratory Syndrome (SARS) epidemic in which people indicated that they would be willing to accept isolation and quarantine in order to help stop the spread of the disease to others [35]. Indeed, it has been suggested that altruistic appeals not only can lead to acceptance of mitigation behaviors like social isolation, but may even prevent long-term negative consequences, including post-traumatic stress disorder [36], that may continue to affect some people long after the pandemic is over.

## Conclusions

Taken together, the present findings suggest that a person’s age, financial status, their levels of anxiety and depression, and their concern for the effects of COVID-19 on others are all important determinants of the extent to which they will adjust their behavior to mitigate the risks of the pandemic. The role of age is of special interest because of the vulnerabilities of older adults, but fortunately it appears to increase both the likelihood and the magnitude of adaptive changes in mitigation behaviors, thereby reducing the risks of the pandemic for the older portion of the population. However, age-related differences in close proximity encounters were not observed in those with scores below cutoffs for anxiety and depressive disorders. Thus, age differences in the frequency of close proximity encounters may be due to age-related decreases in anxiety and depression.

With respect to public health messaging, there is a fine line to be walked between motivating behavior change with realistic risk assessments and creating levels of anxiety or even a sense of hopelessness, both of which may be exacerbated by a climate of mixed and sometimes counter-productive messages. The present findings suggest that framing mitigation in terms of the benefits to others could help in walking that fine line in communicating realistic risks while minimizing the use of fear messages, which appear to carry their own risks. In addition, efforts to reduce psychological stress, worthwhile in their own right, may have the beneficial side effect of increasing adaptive responses to the risks created by the COVID-19 pandemic. Finally, the correlations among mitigation behaviors and the finding that the variables associated with individual mitigation behaviors are also associated with the number of such behaviors that showed adaptive, CDC-recommended changes suggests that rather than affecting a single mitigation behavior, efforts to improve people’s psychological state or their financial situation may efficiently increase the likelihood of changes in multiple mitigation behaviors.

## Supporting information

S1 Questionnaire Pandemic questionnaire. (DOCX)
