# Supplementary material for: Individual differences in COVID-19 mitigation behaviors: The roles of age, gender, psychological state, and financial status
Source: PLoS One. 2021 Sep 21;16(9):e0257658. doi: 10.1371/journal.pone.0257658 (PMC8454939; doi:10.1371/journal.pone.0257658)
Supplement: S1 Questionnaire — (DOCX) [file pone.0257658.s001.docx]

**Self-rated Health**

At the present time, my overall health is:

*Excellent, very good, good, fair, poor.*

**Pandemic Questionnaire**

**Part 1.**

1a. On average this week, how often were you in close proximity (i.e., closer than 6 feet) with people other than those you live with?

*Never (1), once (2), every several days (3), daily (4), more than once daily (5)*

1b. Several months ago, on average how often were you in close proximity (i.e., closer than 6 feet) with people other than those you live with?

*Never (1), once (2), every several days (3), daily (4), more than once daily (5)*

2a. On average, how often did you clean your hands with hand sanitizer or soap and water this week?

*At least once an hour (1), several times a day (2), daily (3), a few times a week (4), not at all (5)*

2b. How often did you usually clean your hands with hand sanitizer or soap and water several months ago?

*At least once an hour (1), several times a day (2), daily (3), a few times a week (4), not at all (5)*

3a. On average, how often this week did you have physical contact (i.e., actual touching) with people other than those you live with?

*Never (1), once (2), every several days (3), daily (4), more than once daily (5)*

3b. Several months ago, how often on average did you have physical contact (i.e., actual touching) with people other than those that you live with?

*Never (1), once (2), every several days (3), daily (4), more than once daily (5)*

4a. This week, on average, how often did you or someone in your household clean and disinfect frequently touched surfaces, such as tables, doorknobs, light switches, countertops, handles, desks, phones, keyboards, toilets, faucets, and sinks)?

*Not at all (1), every several days (2), every other day (3), daily (4), more than once daily (5)*

4b. Several months ago, how often on average did you or someone in your household clean and disinfect frequently touched surfaces?

*Not at all (1), every several days (2), every other day (3), daily (4), more than once daily (5)*

**Part 2.**

5a. How concerned are you about how the coronavirus might affect you personally?

*Not at all (1), a little concerned (2), somewhat concerned (3), concerned (4), very concerned (5)*

5b. How concerned are you about how the coronavirus might affect others?

*Not at all (1), a little concerned (2), somewhat concerned (3), concerned (4), very concerned (5)*

**Part 3.**

6a. Please imagine a ladder with steps numbered from zero at the bottom to ten at the top. Now imagine that the top of the ladder represents the best possible life for you and the bottom of the ladder represents the worst possible life for you.

*If the top step is 10 and the bottom step is 0, on which step of the ladder do you feel you personally stand at the present time**?*

6b. How do you think you would have answered this question two months ago?

*On which step would you have been standing?* *(Again, 10 represents the best possible life for you and 0 represents the worst.)*

6c. How do you think you would answer this question two months from now?

*On which step would you have been standing? (Again, 10 represents the best possible life for you and 0 represents the worst.)*

6d. Finally, how do you think you would answer this question two years from now?

*On which step would you have been standing? (Again, 10 represents the best possible life for you and 0 represents the worst.)*
